# First trans-diagnostic experiences with a novel micro-choice based concentrated group rehabilitation for patients with low back pain, long COVID, and type 2 diabetes: a pilot study

**DOI:** 10.1186/s12916-023-03237-3

**Published:** 2024-01-11

**Authors:** Gerd Kvale, Eirik Søfteland, Marte Jürgensen, Ane Wilhelmsen-Langeland, Anne Haugstvedt, Sigurd William Hystad, Øystein Theodor Ødegaard-Olsen, Bernt Bøgvald Aarli, Sidsel Rykken, Bente Frisk

**Affiliations:** 1 Division of Psychiatry, Haukeland University Hospital, Bergen, Norway; 2 Department of Clinical Psychology, University of Bergen, Bergen, Norway; 3 Helse i Hardanger, Kvam, Norway; 4 Department of Medicine, Haukeland University Hospital, Bergen, Norway; 5 Department of Clinical Science, University of Bergen, Bergen, Norway; 6 Department of Health and Caring Sciences, Western Norway University of Applied Sciences, Bergen, Norway; 7 Department of Psychosocial Science, University of Bergen, Bergen, Norway; 8 Department of Thoracic Medicine, Haukeland University Hospital, Bergen, Norway; 9 Department of Health and Functioning, Western Norway University of Applied Sciences, Bergen, Norway

**Keywords:** Micro-choice, Chronic illness, Concentrated rehabilitation, Low back pain, Post-COVID-19 symptoms, Post COVID-19 condition, Long COVID, Fatigue, Type 2 Diabetes, Anxiety, Depression

## Abstract

**Background:**

The health care is likely to break down unless we are able to increase the level of functioning for the growing number of patients with complex, chronic illnesses. Hence, novel high-capacity and cost-effective treatments with trans-diagnostic effects are warranted. In accordance with the protocol paper, we aimed to examine the acceptability, satisfaction, and effectiveness of an interdisciplinary micro-choice based concentrated group rehabilitation for patients with chronic low back pain, long COVID, and type 2 diabetes.

**Methods:**

Patients with low back pain > 4 months sick-leave, long COVID, or type 2 diabetes were included in this clinical trial with pre-post design and 3-month follow-up. The treatment consisted of three phases: (1) preparing for change, (2) the concentrated intervention for 3–4 days, and (3) integrating change into everyday life. Patients were taught and practiced how to monitor and target seemingly insignificant everyday micro-choices, in order to break the patterns where symptoms or habits contributed to decreased levels of functioning or increased health problems. The treatment was delivered to groups (max 10 people) with similar illnesses. Client Satisfaction Questionnaire (CSQ-8)) (1 week), Work and Social Adjustment Scale (WSAS), Brief Illness Perception Questionnaire (BIPQ), and self-rated health status (EQ-5D-5L) were registered at baseline and 3-month follow-up.

**Results:**

Of the 241 included participants (57% women, mean age 48 years, range 19–84), 99% completed the concentrated treatment. Treatment satisfaction was high with a 28.9 (3.2) mean CSQ-8-score. WSAS improved significantly from baseline to follow-up across diagnoses 20.59 (0.56) to 15.76 (0.56). BIPQ improved from: 22.30 (0.43) to 14.88 (0.47) and EQ-5D-5L: 0.715 (0.01) to 0.779 (0.01)), all *P*<0.001.

**Conclusions:**

Across disorders, the novel approach was associated with high acceptability and clinically important improvements in functional levels, illness perception, and health status. As the concentrated micro-choice based treatment format might have the potential to change the way we deliver rehabilitation across diagnoses, we suggest to proceed with a controlled trial.

**Trial registration:**

ClinicalTrials.gov NCT05234281

**Supplementary Information:**

The online version contains supplementary material available at 10.1186/s12916-023-03237-3.

## Background

The prevalence and cost of chronic diseases are rapidly growing and the trend will continue, not only due to an aging population, but also due to an increasing burden among younger age groups [1, 2]. In this challenging situation, there is an urgent need to develop ways to deliver acceptable and cost-effective treatment approaches that can improve the patients’ functional level, reduce health care utilization and — if suggested by medical guidelines — decrease patients’ need for medications.

Across disorders, medical advice for chronic health challenges typically encompasses recommendations to gradually increase the activity level, while at the same time not overdo it [3–5]. As the main concern for the patient is to prevent the condition from worsening, there is a high risk of developing defensive coping strategies that might contribute to conserve or, in some instances, even exacerbate the problem [6, 7].

Based on existing treatment guidelines [3–5], we have developed a novel approach to deliver interdisciplinary group intervention for chronic health illnesses [8, 9]. One of the main features is a shift in focus, from targeting symptoms to targeting and monitoring seemingly mundane everyday micro-choices that facilitate increased levels of functioning [8]. The intention of these micro-choices is to break unhelpful patterns of symptom regulation by “doing something different” whenever tempted to be guided by the symptoms or habits, with the goal of increasing flexibility and functioning.

The intervention has been delivered to patients with a disparate selection of complex health challenges, namely chronic low back pain, long COVID, type 2 diabetes, and mixed anxiety and depression. The results for patients with anxiety and depression have already been published [10]. These illnesses were chosen as they collectively represent major personal and societal costs, together constituting a large proportion of conditions leading to impaired work participation [11]. Furthermore, they are characterized by fundamentally different symptoms and challenges (e.g., pain, fatigue, depression, anxiety, dyspnea, and hyperglycemia). In consequence, we are able to summarize the overall experience with the intervention across disorders, in addition to the illness-specific outcomes, which will be reported in separate papers.

The aims of this pilot study, detailed in the published protocol paper [8], were to explore the acceptability, satisfaction, and effectiveness of concentrated treatment, as well as changes in illness perception and functional impairment following the intervention, in patients with low back pain, long COVID and type 2 diabetes. In addition, changes in the EuroQoL 5L – health-related quality of life (EQ-5D-5L) were included as an exploratory endpoint. Based on our experiences with other concentrated treatment formats, including anxiety and depression, we hypothesized the intervention to be highly acceptable and to have significant effects on functional impairment [12–16].

## Methods

This study was part of the “Project Development of Smarter Health Solutions” (PUSH project), a collaboration between Haukeland University Hospital (Bergen, Norway) and Helse i Hardanger (Kvam, Norway). The overall aim of the PUSH project was to pilot this novel intervention, and if promising proceed to a controlled trial [8].

### Study design and participants

In this open non-randomized pilot study with a 3-month follow-up design, patients with chronic low back pain, long COVID, and type 2 diabetes were included. The study had a pre-post design, with baseline levels as comparators. The intervention was carried out by an interdisciplinary team (medical doctors, nurses, physical therapists, chiropractors, pharmacists, psychologists, and clinical nutritionists) during 3–4 consecutive days. All included patients had a severity or complexity of their disorder that required health care delivered by relevant medical specialists. General practitioners in the uptake area were informed about the project and were eligible to refer patients to the relevant departments at Haukeland University Hospital. If the patients’ symptoms after initial standard evaluation by the hospital intake team in the given department were considered relevant and severe enough to grant them treatment as a part of public specialist health care, they were screened for participation in the project by a structured short telephone-interview (typically lasting 10 minutes). For inclusion and exclusion criteria, refer to the protocol paper [8]. In short, the most important transdiagnostic eligibility criteria were: oral and written Norwegian fluency, cognitive competency, access to a smartphone, negative COVID-19 polymerase chain reaction test, no severe mental health problems, and/or ongoing uncontrolled substance abuse. Further, for low back pain: no radiculopathy, age 18–70 years, duration > 3 months, and at least 4 months of sick leave within the last year. For long COVID: persistence of symptoms for at least 3 months after the initial infection, lasting for a minimum 2 months, with no other alternative diagnosis to explain these symptoms, no indication of spontaneous recovery, impaired ability to work full time, age 18–67 years. For the diabetes group: confirmed type 2 diabetes mellitus, age >18 years, presence of at least one complicating condition (dysglycemia, hypoglycemias, weight gain, diabetes complications, concrete challenges pertaining to diet, physical activity, and/or medical treatment).

### Procedures and patient flow

**Fig. 1:**
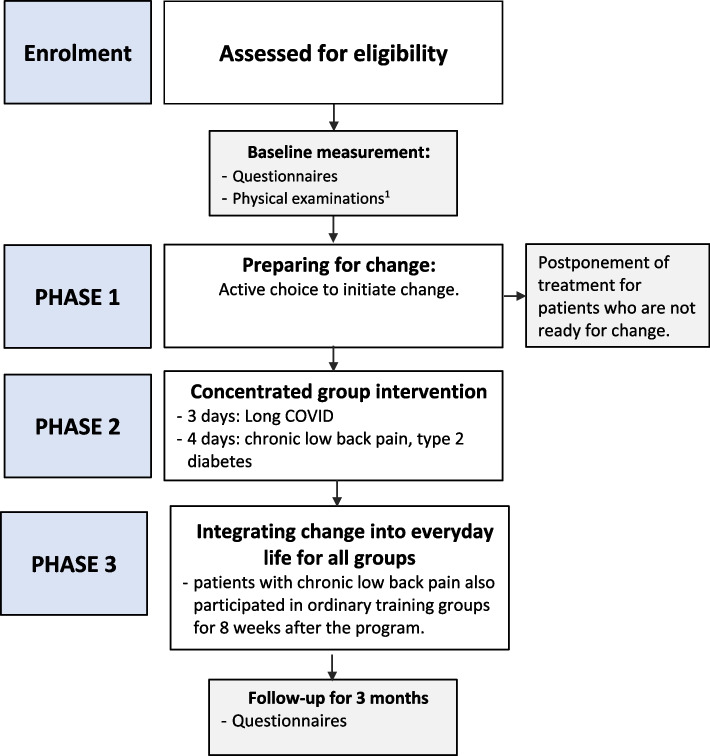
Flowchart of the study. ^1^ There are specific physical exercise test and examinations for the different treatment group, for further details refer to the protocol paper^8^

The treatment was delivered in disease-specific groups of 6–10 patients. See Fig. 1 for an overview of the patient flow and study flowchart. All participants signed an informed consent prior to participation in the study.

### Outline and content of the intervention

A more detailed description of the procedures has already been published [8]. In short, the approach consisted of three phases: (1) preparing for change, (2) the concentrated intervention, (3) integrating change into everyday life. During phase one, the patients had one or two consultations with a member of the interdisciplinary team with a focus on making an active choice of participation in the treatment and in their own change project, in addition to clinical examinations. Prior to the treatment, the patients were informed orally as well as in writing about the program and asked to watch a video describing the aim and content [17]. They also participated in a 1-h physical or digital meeting 1–2 weeks prior to the treatment. In this meeting, it was explained that the aim of the intervention was to increase the level of functioning by targeting all seemingly unimportant behavioral adaptations (“micro-choices”) they had made to deal with their health challenges and to explore new strategies for symptom regulation, guided by the interdisciplinary team. All patients were contacted by a member of the team 1 week prior to the treatment to confirm that they had received all the necessary information and were ready to start their concentrated rehabilitation. The treatment (phase two) was delivered during 3–4 consecutive days and consisted of brief sessions of patient education interspaced with practical sessions where the patients aimed to identify moments of symptom regulation (when the habits or symptoms were making choices “on their behalf”) and to address these by making micro-choices that increased flexibility and their level of functioning. After the practical sessions, the patients shared their experiences with the micro-choice approach with the group, directed by the group leader. Minor disorder-specific adaptations of this principle were made. Participants practiced the method coached by the interdisciplinary team, securing immediate feedback and encouragement. Towards the end of phase two, the patients had to make concrete follow-up plans focusing on integrating the changes into everyday life (phase three). The first 3 weeks after the intervention, patients were daily invited to report online to which extent they were using the new approach towards symptom regulation (these data will be reported separately). Also, the group leader called the patients 10 days after the treatment in order to repeat the core elements of the intervention (doing micro-choices that increase the flexibility and level of functioning).

### Outcomes

Assessments were conducted before and 1 week after the concentrated rehabilitation program, and at 3-month follow-up.

#### Outcome measures

##### Acceptability

The acceptability of the treatment was measured by the following variables: (1) The proportion of patients who accepted participation in the treatment out of those fulfilling inclusion criteria, (2) the proportion of patients who accepted participation that actually started the treatment, and (3) the proportion completing the on-site treatment program.

##### Client Satisfaction Questionnaire (CSQ-8)

The Client Satisfaction Questionnaire (CSQ-8) consists of eight items and is a measure of patient satisfaction with the treatment. Results are rated from 1 (very low satisfaction) to 4 (very high satisfaction). A sum score (8-32) is obtained by summing the item scores [18].

##### Brief Illness Perception Questionnaire (BIPQ)

The Brief Illness Perception Questionnaire (BIPQ) is a nine-item questionnaire designed to assess cognitive and emotional representations of illness [19]. Questions are graded from 1 to 10, with higher scores indicating a greater perceived psychological burden of illness. A sum score (range 0–80) can be calculated by adding together the score of the first eight questions. The scale has good psychometric properties according to a recent review [20]. In line with the published protocol paper, we hypothesized that there would be changes to four of the BIPQ items (see Table 2 – entitled BIPQ_1_) — but not to the complete questionnaire (entitled BIPQ_2_).

##### Work and Social Adjustment Scale (WSAS)

The Work and Social Adjustment Scale (WSAS) consists of 8 items measuring the impact of the illness on aspects of work and social activities [21]. Scores are on a scale from 0 (not at all) to 8 (very severely) with higher scores indicating higher impairment. A sum score (0–40) is calculated by summing the item scores [21].

##### EQ-5D-5L

The EQ-5D-5L includes five items measuring the patients’ self-rated health status within five dimensions: mobility, self-care, everyday activities, pain/discomfort, and anxiety/depression [22]. The items are reported on a 5-level scale from no problem to extreme problems, with higher scores indicating worse levels. A summary index can be derived by weighting each of the levels in each dimension by appropriate national values (i.e., a national value set), where a Norwegian population norm was used [23, 24]. Additionally, the patients were asked to grade their present health status on a visual analog (VAS) scale, where the worst health imaginable would be indicated by 0, whereas the best health as 100.

### Statistical analyses

The statistical analyses were performed in line with the published protocol paper [8]. Between-group differences in the CSQ-8 were analyzed by analysis of variance (ANOVA), using the individual variables (including total score) as responses, and the illness group as factors. Mixed-effects regression models were used to compare WSAS, EQ-5D-5L index, and EQ VAS from pre-treatment to 3-month follow-up, and to compare BIPQ_1_ and BIPQ_2_ across the three assessment points (pre-, 1 week after the intervention, and 3-month follow-up). All participants were included in the analyses, irrespective of missing data at any of the assessment points. Changes within the five EQ dimensions were investigated using McNemar’s tests. Effect sizes of change over time were calculated using Glass’s Δ, with pre-treatment *SD* as the denominator, and were computed using complete data. An effect size is commonly interpreted as small (0.2), moderate (0.5), and large (0.8). Glass’s Δ is the recommended effect size for intervention studies in which there are reasons to believe that the treatment will influence the standard deviation as well as the mean [25]. For all analyses, the statistical significance level was set as *P* < 0.05. All analyses were conducted using Stata version 17.0 (StataCorp LLC, College Station, TX).

### Ethical considerations and data protection

The project, as well as data protection and handling, were approved by the local research ethics board (REK Vest 2020/101638) and were conducted in accordance with the Helsinki Principles. The study was registered in Clinical Trials (NCT05234281, approval date: 05/26/2021). All data were collected through an encrypted application, and anonymized data was transferred to encrypted, access-controlled research server at Helse Vest IKT.

## Results

### Acceptability

Overall, 251 patients who fulfilled the inclusion criteria were offered participation in the rehabilitation program, of whom 96% (241) accepted (range between conditions 94–100%). Furthermore, all included participants attended the concentrated intervention. Finally, 99% (239 participants) completed the on-site concentrated intervention (range 97–100%).

### Demography and baseline characteristics

In total, we included 241 participants, 57% women. Gender was balanced within the disorders, except for long COVID, where 83% were women. Overall, 104 patients with low back pain, 76 with long COVID, and 61 with type 2 diabetes participated. The mean age was 48 years (range 19–84) and was higher for the patients with type 2 diabetes compared to patients with low back pain and long COVID (62 vs. 44 and 41 years, respectively). The mean body mass index was higher in the type 2 diabetes group compared to low back pain and long COVID (30.1 vs 28.0 and 26.5 kg/m^2^). In terms of work participation, 74.8% of the low back patients were on sick leave or disability allowances, correspondingly 53.9% of the long COVID and 27.8% of the diabetes patients. However, 27.0% of the latter were retired from work, which did not feature in the other two groups.

### Satisfaction

The mean CSQ-8 sum score was 28.9 (SD 3.2). No single dimension had an average score ≤ 2, and overall and 97% scored 3 or 4 (Table 1).

**Table 1:** Client satisfaction questionnaire

Item	Low back pain	Long COVID	Diabetes type 2
*1. How would you rate the quality of service you received?*	3.6 (0.5)	3.7 (0.5)	3.7 (0.5)
*2. Did you get the kind of service you wanted?*	**3.4 (0.6)**	**3.4 (0.5)**	**3.7 (0.5)**
*3. To what extent has our program met your needs?*	**3.3 (0.6)**	**3.3 (0.6)**	**3.5 (0.5)**
*4. If a friend were in need of similar help, would you recommend our program to him or her?*	3.8 (0.4)	3.8 (0.4)	3.9 (0.4)
*5. How satisfied are you with the amount of help you received?*	3.6 (0.5)	3.6 (0.5)	3.8 (0.5)
*6. Have the services you received helped you to deal more effectively with your problems?*	**3.5 (0.6)**	**3.6 (0.5)**	**3.8 (0.4)**
*7. In an overall, general sense, how satisfied are you with the service you received?*	3.6 (0.6)	3.7 (0.5)	3.8 (0.4)
*8. If you were to seek help again, would you come back to our program?*	**3.5 (0.5)**	**3.7 (0.5)**	**3.8 (0.4)**
*Total score (possible range is 8-32)*	**28.3 (3.3)**	**28.8 (3.2)**	**29.8 (2.6)**

Data are means (± SD), results in bold indicate *P* < 0.05 for a between-group difference. Item scores range from 1 to 4, with higher scores indicating higher satisfaction

### Clinical outcomes

The mixed regressions analyses showed that level of functioning measured by WSAS improved at follow-up for chronic low back pain (*b* = −4.06, *Z* = 5.19, *P* < .001) and long COVID patients (*b* = −7.66, *Z* = 8.37, *P* < .001), but not for diabetes patients (*b* = −1.88, *Z* = 1.58, *p* = .11). The results further showed that all the patient groups statistically significantly decreased their scores on the hypothesized BIPQ_1_-items from baseline to 1 week after the intervention (Table 2). The scores remained stable at 3-month follow-up except for long COVID patients who had a statistically significant further improvement from 1 week to 3-month follow-up (*b* = −1.85, *Z* = 2.38, *P* = .02). Although not expected, all patient groups also decreased their scores on the remaining BIPQ-items from baseline to 1 week after the intervention and scores remained stable at 3-month follow-up.

**Table 2:** Degree of work and social functioning (WSAS) and illness perceptions (BIPQ) at pre-treatment, post-treatment, and 3-month follow-up

	Pre	Post	Follow-up (FU)	*Χ* ^2^(*df*)	*p*	ES pre-post_†_	ES pre-FU_†_
WSAS							
All patients	20.59 (0.56)_a_		15.76 (0.56)_b_	76.51 (1)	< .001	—	0.61
Type 2 diabetes	12.39 (1.30)_a_		10.51 (1.12)_a_	2.50 (1)	.11	—	0.27
Chronic low back pain	21.53(0.78)_a_		17.48 (0.85)_b_	26.92 (1)	< .001	—	0.45
Long COVID	24.38 (1.00)_a_		16.72 (0.98)_b_	70.13 (1)	< .001	—	1.04
BIPQ_1_							
All patients	22.30 (0.43)_a_	16.32 (0.42)_b_	14.88 (0.47)_c_	256.57 (2)	< .001	1.02	1.35
Type 2 diabetes	18.51 (0.83)_a_	12.57 (0.77)_b_	12.34 (0.80)_b_	61.39 (2)	< .001	0.98	1.00
Chronic low back pain	23.44 (0.67)_a_	19.06 (0.66)_b_	17.63 (1.06)_b_	49.66 (2)	< .001	0.79	1.02
Long COVID	24.55 (0.72)_a_	16.72 (0.73)_b_	14.86 (0.70)_c_	175.64 (2)	< .001	1.52	1.89
BIPQ_2_							
All patients	20.79 (0.38)_a_	18.18 (0.37)_b_	17.70 (0.42)_b_	63.63 (2)	< .001	0.51	0.85
Type 2 diabetes	19.19 (0.73)_a_	17.31 (0.68)_b_	17.60 (0.71)_b_	7.20 (2)	< .05	0.35	0.31
Chronic low back pain	23.21 (0.59)_a_	21.74 (0.58)_b_	20.67 (0.92)_b_	10.01 (2)	< .01	0.29	.59
Long COVID	19.94 (0.64)_a_	15.48 (0.64)_b_	15.07 (0.63)_b_	66.79 (2)	< .001	1.10	1.24

Estimated means and standard errors from mixed regressions are presented

*WSAS* The Work and Social Adjustment Scale, *BIPQ* Brief Illness Perception Questionnaire, *FU* 3-month follow-up after intervention, *ES* effect size computed as $\mathrm G\mathrm l\mathrm a\mathrm s\mathrm s'\mathrm s\;\mathrm\Delta=\frac{M_{pre}-M_{post}}{{SD}_{pre}}$. Different subscripts (^a, b, c^) within a row indicate statistically significant differences between means

_BIPQ1_Includes the BIPQ items “How much does your illness affect your life?”, “How much control do you feel you have over your illness?”, “ How concerned are you about your illness?”, and “How well do you feel you understand your illness?”

_BIPQ2_Also includes the BIPQ items “How long do you think your illness will continue?”, “How much do you experience symptoms from your illness?”, “How much do you think your treatment can help your illness?”, and “How much does your illness affect you emotionally?”

^†^Effect sizes are computed based on complete data

As for EQ-5D-5L, from baseline to 3-month follow-up, there were statistically significant improvements for mobility, usual activities, and anxiety/depression, but not for self-care or pain/discomfort (Table 3). Further, the EQ-5D-5L index improved for all groups except diabetes, who scored equal to the national Norwegian population norm (Table 4) [24]. Significant improvements were also found in terms of self-perceived general health (VAS scale). The ten most commonly reported health states at baseline can be found in Additional file 1.

**Table 3:** Distribution of EQ-5D-5L dimensions at baseline and at follow-up

	All Patients		Type 2 diabetes		Chronic low back pain		Long COVID	
	Baseline (*n*/%)	3 mos. fu. (*n*/%)	Baseline (*n*/%)	3 mos. fu. (*n*/%)	Baseline (*n*/%)	3 mos. fu. (*n*/%)	Baseline (*n*/%)	3 mos. fu. (*n*/%)
*Mobility*								
No problem	102 (50.3)	120 (59.1)	41 (69.5)	45 (76.3)	22 (24.7)	37 (41.6)	39 (70.9)	38 (69.1)
Any problem	101 (49.7)	83 (40.9)	18 (30.50)	14 (23.7)	67 (75.3)	52 (58.4)	16 (29.1)	17 (30.9)
χ^2^	6.23		2.67		7.26		0.07	
*p* ^a^	.012		.219		.011		1	
*Self-care*								
No problem	164 (80.8)	170 (83.7)	58 (98.3)	57 (96.6)	54 (60.7)	62 (69.7)	52 (94.6)	51 (92.7)
Any problem	39 (19.2)	33 (16.3)	1 (1.7)	2 (3.4)	35 (39.3)	27 (30.3)	3 (5.4)	4 (7.3)
χ^2^	1.12		.033		2.91		0.14	
*p* ^a^	.377		1		.134		1	
*Usual activities*								
No problem	49 (24.1)	86 (42.4)	40 (67.8)	44 (74.6)	3 (3.4)	20 (22.5)	6 (10.9)	22 (40)
Any problem	154 (75.9)	117 (57.6)	19 (32.2)	15 (25.4)	86 (96.6)	69 (77..5)	49 (89.1)	33 (60)
χ^2^	22.44		0.89		15.21		10.67	
*p* ^a^	< .001		.481		< .001		.002	
*Pain/discomfort*								
No problem	19 (9.4)	24 (11.8)	12 (20.3)	15 (25.4)	0 (0)	1 (1.1)	7 (12.7)	8 (14.6)
Any problem	184 (90.6)	179 (88.2)	47 (79.7)	44 (74.6)	89 (100)	88 (98.9)	48 (87.3)	47 (85.4)
χ^2^ (df)	1.09		0.69		1		0.11	
*p* ^a^	.405		.581		1		1	
Anxiety/depression								
No problem	85 (41.9)	99 (48.8)	37 (62.7)	37 (62.7)	33 (37.1)	41 (46.1)	15 (27.3)	21 (38.2)
Any problem	118 (58.1)	104 (51.2)	22 (37.3)	22 (37.3)	56 (62.9)	48 (53.9)	40 (72.7)	34 (61.8)
χ^2^ (df)	4.26		0.00		2.91		2.25	
*p* ^a^	.054		1		.134		.210	

The EQ-5D levels were dichotomised into “no problems” (level 1) and “any problems” (levels 2–5)

^a^McNemar’s exact significance probability

**Table 4:** Estimated means (SE) on EQ-5D-5L index and VAS from mixed regressions at pre-treatment and 3-month follow-up

	Baseline	Follow-up	*Z*	*p*	ES
EQ-5D index					
All patients	0.715 (0.01)	0.779 (0.01)	6.23	< .001	0.38
Type 2 diabetes	0.842 (0.02)	0.859 (0.02)	0.95	.342	0.15
Chronic low back pain	0.621 (0.01)	0.725 (0.02)	6.93	< .001	0.54
Long COVID	0.748 (0.02)	0.796 (0.02)	2.45	.014	0.33
Norwegian population norm^23^	0.805				
EQ VAS					
All patients	54.1 (1.03)	62.8 (1.15)	6.45	< .001	0.53
Type 2 diabetes	65.8 (2.08)	69.1 (2.14)	1.32	.185	0.23
Chronic low back pain	47.4 (1.51)	56.7 (1.73)	4.70	< .001	0.58
Long COVID	54.2 (1.86)	67.1 (2.19)	5.14	< .001	0.78
Norwegian population norm^23^	77.9				

*ES* Effect size computed as $\mathrm G\mathrm l\mathrm a\mathrm s\mathrm s'\mathrm s\;\mathrm\Delta=\frac{M_{pre}-M_{post}}{{SD}_{pre}}$

## Discussion

The concentrated micro-choice focused intervention was highly acceptable with a 99% on-site completion rate. Further, all the CSQ-8 dimensions were in line with excellent patient satisfaction. Finally, clinically meaningful improvements in the level of functioning and how much the illness affected the patients’ lives, were also achieved.

This novel approach to delivering concentrated evidence-based rehabilitation to highly challenging groups of patients suffering from a broad range of complex, chronic disorders was associated with high satisfaction with the extent of the treatment, in spite of our approach being substantially shorter than traditional 3–4-week rehabilitation interventions. Similarly, across disorders, patients were highly satisfied with the amount of help, indicating that their needs were met. This could imply that a concentrated approach could be a more cost- and time-effective way of delivering rehabilitation, not only from the perspective of health economics, but also for the time invested by the individual participants.

Several aspects of the intervention – detailed in the protocol paper – break with the typical mode of rehabilitation: e.g., (1) distinct phases, including a separate pretreatment preparation for change, (2) the concentrated format (3–4 consecutive days), (3) a shift in focus from symptoms to actions (indicating that change is within reach, installing hope in the patients), (4) focusing on the myriad everyday opportunities for “doing something different” than what the habits or symptoms suggest, i.e., the micro-choices, (5) starting to practice breaking unhelpful patterns of symptom regulation in a safe context together with health care professionals, giving the opportunity to correct and modify unhelpful behavior patterns when they occur, and (6) continue practicing in the patients’ every-day life [8].

In the current pilot study, the participants were required to stay near the treatment facility for 3–4 days, which might be a potential limitation for future implementation studies as well as in terms of feasibility. We have now started to deliver the treatment fully digitalized (combination of phone calls, electronic meetings, and app), and it would be highly interesting to compare these two modes of deliverance in a future randomized controlled trial.

The goal of the concentrated rehabilitation was to increase the patients’ level of functioning (WSAS), which overall was achieved for all illnesses, but not in the diabetes group. Type 2 diabetes differs from the other diagnoses in that the health problems are less related to symptoms, but rather more to the complexity of handling the disease (including glycemic control and weight), as well as more subtle and non-specific complaints. This is reflected in a flooring effect i.e., that they did not score poorly on this measure *before* treatment. Further, more participants in the diabetes group were retired, which affects the WSAS results. Overall, the degree of changes in all outcomes (illness perception (BIPQ_1_), functional levels (WSAS) as well as self-perceived health (EQ-5D-5L)) were large, with clinically significant effect estimates. Interestingly, changes were most pronounced for the long COVID group, both at 1 week, and further increasing at 3 months of follow-up, in line with results that we have previously published for long COVID-specific outcomes [26]. Indeed, a recent review emphasized the limited evidence on the impact of rehabilitation approaches for long COVID, specifically in terms of levels of functioning, underscoring the relevance of our findings [27]. For diabetes, although a major public health challenge, no rehabilitation studies focusing on similar outcomes as ours were available for comparison. Looking at rehabilitation studies on low-back pain, these typically focus on the level of symptoms, rather than the impact on illness perception and self-perceived health, although some point towards the importance of such outcomes [28]. In terms of changes to level functioning (WSAS), our effect sizes are comparable or exceed results from studies using other approaches such as physical therapy informed by acceptance and commitment therapy, usual care, or treatment based on the fear-avoidance model [29, 30]. Hence, it seems clear that the outcomes improved trans-diagnostically, in line with what our group also found for patients with anxiety and/or depression [10]. To our knowledge, no similar trans-diagnostic intervention program has been described. Even though the design does not allow for speculations regarding mechanisms for change, it is noteworthy that the change in functional levels followed the shift in symptom regulation where the patients were encouraged to “do something different,” i.e., to increase activity level when symptoms or habits dictated otherwise, instead of trying to reduce the symptoms.” It is important to underscore that, prior to the concentrated treatment, patients were introduced to the main concepts of the approach. This includes the concept of initiating change by breaking the typical patterns of symptom regulation as well as the concept of micro-choices. Furthermore, the importance of making a clear decision to initiate change and the necessity to participate wholeheartedly in order to facilitate and maintain change was emphasized, e.g., “no treatment works if you do not take the medicine.” Patients were encouraged to postpone the treatment if they were not ready to fully engage. In sum, these elements provided the patients with an opportunity to decide and to take responsibility for their own change projects from the get-go. Thus, while being very direct in our approach with regards to informing the patients that improvement could not be expected if they did not fully engage in the treatment, more than 90% of the eligible patients wanted to participate, and nearly all completed and were satisfied, in addition to achieving significantly improved levels of functioning. We further believe that our approach on-site, with long daily sessions where the patients practiced breaking unhelpful patterns side-by-side with other patients, and with health care professionals as competent supervisors giving feedback in real-time, is crucial to the positive results. Finally, shifting focus from monitoring symptoms (including pain, fatigue, thought patterns, worries, habits) to deliberate choices and behavior (micro-choices) promotes the idea that individually relevant change is within reach for each individual.

It is also highly interesting to note that a large and significant change was achieved already 1 week after the intervention, which might be surprising given the chronicity of the health challenges. Our results are in line with the already published results for the same intervention in mixed anxiety/depression, and also for previous experiences with this format in obsessive-compulsive disorder, panic disorder, and chronic fatigue syndrome [10, 12, 13, 15, 16, 31]. This could indicate that the micro-choice focused concentrated rehabilitation approach has a potential for substantial generic and transdiagnostic effects. Moving forward, we speculate that a shift from a diagnosis-based intervention to focusing on the handling of the most dominating symptoms (i.e., pain/fatigue) could be useful. Further long-term studies are needed to shed light on this.

Although the concentrated treatment format implies a condensed and potentially highly cost-effective approach compared to traditional formats, this needs to be further investigated. In our opinion, there are potentials for even greater cost-effectiveness, as several elements of the treatment could be delivered digitally to an even larger group of patients. Identifying which patients need a face-to-face approach vs. digitally should be a priority for future research projects.

Finally, our study has several limitations. Although the results were uniformly positive, the non-randomized study design might be vulnerable to selection bias. In this pilot study more than 90% of the eligible patients accepted participation and completed the treatment, limiting the selection bias. Since this was a pre-post design with patients as their own controls, we cannot claim that the improvements were due to the intervention itself. On the other hand, all three conditions are chronic, with a low likelihood of spontaneous improvements. The low back pain patients had at least 4 months of sick leave due to the condition. Other studies have shown that the prognosis for this group is poor [32, 33]. As for the long COVID patients, they were not included if they showed signs of spontaneous recovery in the waiting period. Interestingly, a Norwegian cohort of young adult individuals with mild primary infection, showed that more than half had substantial symptoms of long COVID at 6 months, including 21% with fatigue [34]. Finally, type 2 diabetes is a chronic disorder, where the natural history includes progressive loss of both beta-cell function as well as of quality of life [35]. Consequently, although we must refrain from causal inferences, we argue that the likelihood of the results being spurious — across such various disorders — is low.

## Conclusions

There is a great need for novel, cost-effective rehabilitation approaches to patients with complex chronic illnesses. This concentrated micro-choice focused group-based intervention was highly acceptable to >90% of patients with chronic low back pain, long COVID, and type 2 diabetes. Further, patients were highly satisfied, also with the length of the treatment. At 3-month follow-up, clinically and statistically significant improvements of the level of functioning, as well as the illness perception and self-rated health status were found. The results are in agreement with those from a similar intervention in people with mixed anxiety/depression [10]. Hence, the concentrated micro-choice based intervention could represent a promising generic approach to achieving meaningful improvements in patients with complex chronic conditions.

### Supplementary Information

**Additional file 1.** The Ten Most Frequently reported EQ-5D-5L Health States at Baseline.

## Data Availability

The data that support the findings of this study are available from Youwell A/S, but restrictions apply to the availability of these data, which were used under license for the current study, and so are not publicly available. Data are, however, available from the authors upon reasonable request and with permission of Youwell A/S.

## Abbreviations

ANOVA: Analysis of variance
BIPQ: Brief Illness Perception Questionnaire
CSQ-8: Client Satisfaction Questionnaire-8
EQ-5D-5L: Euro Quality of Life Questionnaire
LOCF: Last Observation Carried Forward
PUSH: Project Development of Smarter Health Solutions
VAS: Visual analog scale
WSAS: Work and Social Adjustment Scale

## References

[1] WHO Noncommunicable diseases. https://www.who.int/health-topics/noncommunicable-diseases#tab=tab_1.

[2] Akseer N, Mehta S, Wigle J, Chera R, Brickman ZJ, Al-Gashm S (2020). Non-communicable diseases among adolescents: current status, determinants, interventions and policies. BMC Public Health..

[3] Oliveira CB, Maher CG, Pinto RZ, Traeger AC, Lin CWC, Chenot JF (2018). Clinical practice guidelines for the management of non-specific low back pain in primary care: an updated overview. Eur Spine J..

[4] WHO. Rehabilitation and COVID-19. https://www.who.int/teams/noncommunicable-diseases/covid-19/rehabilitation.

[5] Care D, Suppl SS. 5. Facilitating Behavior Change and Well-being to Improve Health Outcomes: Standards of Medical Care in Diabetes—2022. Diabetes Care. 2022;45 January:S60–82. 10.2337/dc22-S005 34964866

[6] Carver CS, Scheier MF (1994). Situational coping and coping dispositions in a stressful transaction. J Pers Soc Psychol..

[7] Van den Bergh O, Witthöft M, Petersen S, Brown RJ (2017). Symptoms and the body: taking the inferential leap. Neurosci Biobehav Rev.

[8] Kvale G, Frisk B, Jürgensen M, Børtveit T, Ødegaard-Olsen ØT, Wilhelmsen-Langeland A (2021). Evaluation of novel concentrated interdisciplinary group rehabilitation for patients with chronic illnesses: Protocol for a nonrandomized clinical intervention study. JMIR Res Protoc.

[9] Frisk B, Njøten KL, Aarli B, Hystad SW, Rykken S, Kjosås A (2022). A Novel concentrated, interdisciplinary group rehabilitation program for patients with chronic obstructive pulmonary disease: protocol for a nonrandomized clinical intervention study. JMIR Res Protoc..

[10] Kvale G, Langeland AW, Jürgensen M, Hystad SW, Öst LG. Concentrated transdiagnostic and cross ‑ disciplinary group treatment for patients with depression and with anxiety : a pilot study. BMC Psychiatry. 2022;:1–11. 10.1186/s12888-022-04229-y PMC9441319 36058925

[11] NAV. Sick leave and disability statistics. 2023. https://www.nav.no/no/nav-og-samfunn/statistikk/sykefravar-statistikk/sykefravar.

[12] Hansen B, Hagen K, Öst L-G, Solem S, Kvale G (2018). The Bergen 4-day OCD treatment delivered in a group setting: 12-month follow-up. Front Psychol..

[13] Stubhaug B, Lier HO, Aßmus J, Rongve A, Kvale G (2018). A 4-Day Mindfulness-Based Cognitive Behavioral Intervention Program for CFS/ME. An Open Study, With 1-Year Follow-Up. Front Psychiatry.

[14] Hansen B, Kvale G, Hagen K, Havnen A, Öst L-G (2019). The Bergen 4-day treatment for OCD: four years follow-up of concentrated ERP in a clinical mental health setting. Cogn Behav Ther..

[15] Kvale G, Hansen B, Björgvinsson T, Børtveit T, Hagen K, Haseth S (2018). Successfully treating 90 patients with obsessive compulsive disorder in eight days: the Bergen 4-day treatment. BMC Psychiatry..

[16] Launes G, Hagen K, Sunde T, Öst L-G, Klovning I, Laukvik I-L (2019). A Randomized Controlled Trial of Concentrated ERP, Self-Help and Waiting List for Obsessive- Compulsive Disorder: The Bergen 4-Day Treatment. Front Psychol.

[17] Helse i Hardanger (introduction film).2021. https://www.youtube.com/watch?v=qHW0IIlW_QM&t=124s

[18] Nguyen TD, Attkisson CC, Stegner BL (1983). Assessment of patient satisfaction: development and refinement of a service evaluation questionnaire. Eval Program Plann.

[19] Broadbent E, Petrie KJ, Main J, Weinman J (2006). The brief illness perception questionnaire. J Psychosom Res..

[20] Broadbent E, Wilkes C, Koschwanez H, Weinman J, Norton S, Petrie KJ (2015). A systematic review and meta-analysis of the brief illness perception questionnaire. Psychol Heal..

[21] Mundt JC, Marks IM, Shear MK, Greist JH (2002). The work and social adjustment scale: a simple measure of impairment in functioning. Br J Psychiatry.

[22] Herdman M, Gudex C, Lloyd A, Janssen M, Kind P, Parkin D (2011). Development and preliminary testing of the new five-level version of EQ-5D (EQ-5D-5L). Qual Life Res..

[23] Devlin N, Roudijk B, Ludwig K (2022). Value Sets for EQ-5D-5L.

[24] Garratt AM, Hansen TM, Augestad LA, Rand K, Stavem K (2022). Norwegian population norms for the EQ-5D-5L: results from a general population survey. Qual Life Res..

[25] Lakens D (2013). Calculating and reporting effect sizes to facilitate cumulative science: a practical primer for t-tests and ANOVAs. Front Psychol.

[26] Frisk B, Jürgensen M, Espehaug B, Njøten KL, Søfteland E, Aarli BB, et al. OPEN A safe and effective micro ‑ choice based rehabilitation for patients with long COVID : results from a quasi ‑ experimental study. Sci Rep. 2023;:1–10. 10.1038/s41598-023-35991-y PMC10252160 37296140

[27] Davis HE, McCorkell L, Vogel JM, Topol EJ (2023). Long COVID: major findings, mechanisms and recommendations. Nat Rev Microbiol.

[28] Garratt AM, Furunes H, Hellum C, Solberg T, Brox JI, Storheim K (2021). Evaluation of the EQ-5D-3L and 5L versions in low back pain patients. Health Qual Life Outcomes..

[29] Ryum T, Stiles TC (2023). Changes in pain catastrophizing, fear-avoidance beliefs, and pain self-efficacy mediate changes in pain intensity on disability in the treatment of chronic low back pain. PAIN Reports..

[30] Godfrey E, Wileman V, Galea Holmes M, McCracken LM, Norton S, Moss-Morris R (2020). Physical therapy informed by acceptance and commitment therapy (PACT) versus usual care physical therapy for adults with chronic low back pain: a randomized controlled trial. J Pain..

[31] Hansen B, Kvale G, Hagen K, Hjelle KM, Solem S, Bø B, et al. The Bergen 4-day treatment for panic disorder: a pilot study. Front Psychol. 2018;9:1044. 10.3389/fpsyg.2018.01044 PMC6029556 29997546

[32] Vibe Fersum K, O’Sullivan P, Skouen JS, Smith A, Kvåle A (2013). Efficacy of classification-based cognitive functional therapy in patients with non-specific chronic low back pain: a randomized controlled trial. Eur J Pain..

[33] Hartvigsen J, Hancock MJ, Kongsted A, Louw Q, Ferreira ML, Genevay S (2018). What low back pain is and why we need to pay attention. Lancet..

[34] Blomberg B, Mohn KGI, Brokstad KA, Zhou F, Linchausen DW, Hansen BA (2021). Long COVID in a prospective cohort of home-isolated patients. Nat Med..

[35] Shamshirgaran SM, Stephens C, Alpass F, Aminisani N (2020). Longitudinal assessment of the health-related quality of life among older people with diabetes: results of a nationwide study in New Zealand. BMC Endocr Disord..

