# Supplementary material for: First trans-diagnostic experiences with a novel micro-choice based concentrated group rehabilitation for patients with low back pain, long COVID, and type 2 diabetes: a pilot study
Source: BMC Med. 2024 Jan 11;22:12. doi: 10.1186/s12916-023-03237-3 (PMC10782659; doi:10.1186/s12916-023-03237-3)
Supplement: Supplementary file 1 — Additional file 1. The Ten Most Frequently reported EQ-5D-5L Health States at Baseline. [file 12916_2023_3237_MOESM1_ESM.docx]

**Additional file 1. The Ten Most Frequently reported EQ-5D-5L Health States at Baseline**

|  | All patients | |  |  | Type 2 diabetes | |  |  | Chronic low back pain | |  |  | Long COVID | |
| --- | --- | --- | --- | --- | --- | --- | --- | --- | --- | --- | --- | --- | --- | --- |
| Health state | *n* | % |  | Health state | *n* | % |  | Health state | *n* | % |  | Health state | *n* | % |
| 11121 | 13 | 4.8 |  | 11121 | 12 | 19.4 |  | 11222 | 3 | 2.5 |  | 11232 | 5 | 5.8 |
| 11122 | 11 | 4.1 |  | 11111 | 8 | 12.9 |  | 11231 | 3 | 2.5 |  | 11221 | 4 | 4.6 |
| 11111 | 9 | 3.4 |  | 11122 | 7 | 11.3 |  | 11331 | 3 | 2.5 |  | 11222 | 4 | 4.6 |
| 11222 | 9 | 3.6 |  | 21221 | 6 | 9.7 |  | 21222 | 3 | 2.5 |  | 11223 | 4 | 4.6 |
| 21221 | 9 | 3.4 |  | 11112 | 3 | 4.8 |  | 21231 | 3 | 2.5 |  | 11332 | 4 | 4.6 |
| 11221 | 7 | 2.6 |  | 11131 | 2 | 3.2 |  | 21234 | 3 | 2.5 |  | 11122 | 3 | 3.5 |
| 11223 | 6 | 2.2 |  | 11222 | 2 | 3.2 |  | 23331 | 3 | 2.5 |  | 11322 | 3 | 3.5 |
| 11231 | 6 | 2.2 |  | 21231 | 2 | 3.2 |  | 32442 | 3 | 2.5 |  | 21322 | 3 | 3.5 |
| 11332 | 5 | 1.9 |  | 11124 | 1 | 1.6 |  | 11221 | 2 | 1.7 |  | 11212 | 2 | 2.3 |
| 21222 | 5 | 1.9 |  | 11141 | 1 | 1.6 |  | 11241 | 2 | 1.7 |  | 11242 | 2 | 2.3 |
